# Sulforaphane alleviates membranous nephropathy by inhibiting oxidative stress-associated podocyte pyroptosis

**DOI:** 10.22038/ijbms.2024.78960.17083

**Published:** 2025

**Authors:** Daoyuan Lv, Laping Chu, Yuan Du, Chunqing Li, Neng Bao, Yuqing Su, Gang Wang, Yanlie Zheng, Yafen Yu

**Affiliations:** 1 Department of Nephrology, Affiliated Hospital of Jiangnan University, Wuxi, China; 2 Wuxi School of Medicine, Jiangnan University, Wuxi, China; 3 Department of Nephrology, Jiangmen Central Hospital, Jiangmen, China; 4 Department of Infectious Disease, General Hospital of Southern Theater Command, Guangzhou, China

**Keywords:** Membranous nephropathy, Oxidative stress, Podocyte, Pyroptosis, Sulforaphane

## Abstract

**Objective(s):**

To investigate the natural product sulforaphane (SFN) in protection of membranous nephropathy (MN) by inhibiting oxidative stress-associated podocyte pyroptosis.

**Materials and Methods:**

A passive Heymann nephritis (PHN) model was established and treated with SFN. Clinical manifestations were examined by testing 24-hr urine protein, albumin, total cholesterol, triglyceride, high-density and low-density lipoprotein levels. Podocyte injury was observed through glomerular ultrastructure and the expression of podocin and desmin. Intrarenal oxidative stress was evaluated through assessment of oxidative markers, including malondialdehyde, 8-isoprostane, and 8-hydroxydeoxyguanosine, and the activities of anti-oxidant enzymes, including total superoxide dismutase, catalase, and γ-glutamylcysteine synthetase. Podocyte and intrarenal pyroptosis were investigated by observing the localization of the GSDMD N-terminus (GSDMD(N)) in podocytes; the expression of pyroptosis signaling pathway, including GSDMD, NF-κB p65, p-NF-κB p65 (Ser536), NLRP3, ASC, caspase-1, IL-1β, and IL-18; and pyroptosis encounter Nrf2 in the glomeruli and kidney.

**Results:**

SFN has a protective effect on MN, as reflected by alleviation of nephrotic syndrome, amelioration of podocyte foot process fusion, increased expression and normalization of podocin, and decreased expression of desmin in the glomeruli. Mechanistically, SFN relieved intrarenal oxidative stress, as indicated by decreased renal malondialdehyde, 8-isoprostane, and 8-hydroxydeoxyguanosine and increased activity of total superoxide dismutase, catalase, and γ-glutamylcysteine synthetase. SFN also inhibited podocyte and intrarenal pyroptosis, as revealed by decreased colocalization of GSDMD (N) with synaptopodin and ZO-1, decreased expression of pyroptosis signaling pathway, and increased expression of Nrf2 in the glomeruli and kidney.

**Conclusion:**

SFN could alleviate MN by inhibiting oxidative stress-associated podocyte pyroptosis.

## Introduction

Membranous nephropathy (MN) is a kidney-specific autoimmune disease, a leading cause of adult nephrotic syndrome, and a common risk factor for end-stage renal disease (1). Recently, the prevalence of MN has increased significantly in China and other developing countries (2-5), imposing a great burden on health. MN is pathologically characterized by the thickening of the glomerular capillary walls and the accumulation of immune deposits on the subepithelial aspect of the glomerular capillary wall, which is close to podocytes (6). Podocyte injury caused by immunological response-induced complement activation plays a central role in renal injury in MN (7). However, the mechanisms underlying podocyte injury in MN remain incompletely understood.

Pyroptosis is a proinflammatory form of regulated necrosis that involves cell membrane pore formation by oligomers of the N-terminal fragment of gasdermins after gasdermin cleavage (8). Oxidative stress is a cellular stress response caused by an imbalance between oxidants and anti-oxidants in favor of oxidants (9). Emerging evidence has suggested the close relationship between oxidative stress and pyroptosis in the pathogenesis of kidney diseases (10). In 2022, Wang *et al.* (11) observed the promoting effect of oxidative stress on podocyte pyroptosis caused by complement *in vitro*, providing new opportunities for anti-oxidant and anti-pyroptosis treatment of MN. Therefore, the protective effects of more anti-oxidants and pyroptosis inhibitors on renal and podocyte injury in MN remain to be elucidated.

Sulforaphane (SFN) is an isothiocyanate found in cruciferous vegetables and is a natural, classic anti-oxidant that has protective effects on the majority of tissues and organs (12). Investigations have revealed the ameliorative effect of SFN on kidney diseases, including acute kidney injury, diabetic nephropathy, and lupus nephritis, due to its anti-oxidative properties (13). Recently, SFN was shown to inhibit the pyroptosis cascade by reducing the activation of NLRP3 inflammasome and inhibiting the activation of caspase-1 and IL-1β after oxidative stress remission (14). Therefore, we hypothesized that SFN could exert renoprotective effects on MN by inhibiting oxidative stress-associated podocyte pyroptosis. The present study used the passive Heymann nephritis (PHN) rat model to validate the renoprotective, anti-oxidative, and antipyroptotic effects of SFN on MN.

## Materials and Methods

***Animals***

Female Sprague-Dawley (SD) rats weighing 150–180 g were obtained from Sino-British SIPPR/BK Lab (Shanghai, China). All rats were housed under standard conditions and fed rat chow and water *ad libitum* in the Laboratory Animal Center of Wuxi School of Medicine, Jiangnan University. The protocol for the animal experiments was approved by the Experimental Animal Ethics Committee of Jiangnan University (Approval number: JN.No20231115S0400630[547]).

***PHN model establishment***

Rabbit anti-Fx1A serum was prepared as previously described (15). In brief, Fx1A was isolated from the kidneys of SD rats by sieving and ultracentrifugation. The rabbit was subcutaneously immunized with Fx1A emulsified in complete Freund’s adjuvant for at least four immunizations, and rabbit anti-Fx1A serum was obtained by plasmapheresis. Anti-Fx1A antibody concentrations were validated according to the immunofluorescence intensity of the rat proximal renal tubular epithelial cell brush border. Rats were given two intraperitoneal injections of anti-Fx1A antiserum in volumes of 2 and 1 ml at 1-hour intervals to establish the PHN model. An equal amount of normal rabbit serum was injected into rats in the normal control (NC) group in the same manner.

***Treatment, groupings, and sample collection***

SFN, which was obtained from MedChemExpress LLC (#HY-13755, Shanghai, China), was dissolved in 10% DMSO+40% PEG300+5% Tween-80+45% normal saline. PHN rats were intraperitoneally injected with 5 mg/kg SFN daily throughout the observation period beginning on the day before model establishment. All rats were randomly divided into three groups: NC, PHN, and PHN+SFN. The observation period was 15 days. Body weight, serum, and 24-hour urine samples were acquired by weighing scale, angular venipuncture, and metabolism cages, respectively, on the 1st, 5th, 8th, and 15th days of the observation period. The rats were euthanized on the 15th day, and the kidneys were collected. One rat from the PHN group died accidentally within the observation period and was discarded, while no rats from the NC and PHN+SFN groups suffered accidental death. Finally, six rats from each group were included for further analysis.

***Urine protein and blood biochemical tests***

Urine proteins (UPro) concentrations were tested using the Bradford method, and the 24 hr-UPro was calculated according to the urine volume. Albumin (ALB), total cholesterol (T-CHOL), triglyceride (TG), high-density lipoprotein (HDL), low-density lipoprotein (LDL), serum creatinine (SCr), and blood urea nitrogen (BUN) levels were tested with an automatic biochemical analyzer (ZY-450, Kehua Bioengineering Co., Ltd., Shanghai, China).

***Histopathology***

Fresh renal cortices were fixed with 10% formaldehyde for at least 6 hr and embedded in paraffin after dehydration and clearing. Then, 2.5-μm-thick paraffin sections were prepared with a microtome. After deparaffinization and rehydration, the sections were subjected to Masson trichrome staining and periodic acid silver methenamine (PASM)-Masson staining. Representative micrographs of the stained tissue were captured with a light microscope (Eclipse 80i, Nikon Instruments Inc., Tokyo, Japan).

***Transmission electron microscopy (TEM)***

Fresh 1 mm^2 ^granules of the renal cortex were fixed with 2.5% glutaraldehyde at 4 °C overnight. The fixed tissue was then washed with PBS and postfixed with 1% osmium tetroxide at room temperature (RT) for 1 hr. Then, 70-nm-thick ultrathin sections were prepared with an ultramicrotome and stained with uranyl acetate and lead citrate. The ultrastructure of the stained tissue was observed, and the average foot process width of podocytes was calculated according to the following formula: (π/4) × (Σ glomerular basement membrane (GBM) length/Σ number of foot processes) by using DigitalMicrograph software (Gatan, Inc., Pleasanton, USA) (16). Representative photomicrographs were obtained with an electron microscope (H-7500, Hitachi, Ltd., Tokyo, Japan).

***Immunofluorescence analysis***

Five μm-thick frozen sections were subjected to immunofluorescence staining. For podocin staining, the sections were blocked with 10% calf serum and incubated with podocin antibodies (1:500 dilution, v/v, #20384-1-AP, Proteintech Group Inc., Rosemont, USA) at RT overnight and subsequently incubated with FITC-labeled secondary antibodies (1:200 dilution, v/v, #A0562, Beyotime Biotechnology, Shanghai, China) at RT for 40 min. For double immunofluorescence staining, the blocked sections were incubated with cleaved N-terminal GSDMD (GSDMD(N)) (1:150 dilution, v/v, #ab215203, Abcam plc., Cambridge, UK) plus synaptopodin (1:500 dilution, v/v, #sc-515842, Santa Cruz Biotechnology, Inc., Dallas, USA) and GSDMD(N) (1:150 dilution, v/v) plus ZO-1 (1:200 dilution, v/v, #66452-1-Ig, Proteintech Group Inc.) antibodies at RT overnight and subsequently incubated with FITC-labeled (1:200 dilution, v/v) and Cy3-labeled secondary antibodies (1:200 dilution, v/v, #A0521, Beyotime Biotechnology) at RT for 40 min. Representative micrographs of the stained tissue were captured with confocal microscopy (LSM710, Carl Zeiss Meditec AG, Oberkochen, Germany).

***Immunohistochemistry***

2.5 μm-thick paraffinized sections were subjected to immunohistochemical staining, and the deparaffinized and rehydrated sections were boiled in Tris-EDTA (pH=8.0) for 10 min for antigen retrieval. After being blocked with 10% calf serum, the sections were incubated with the following antibodies at RT overnight: anti-Desmin (#MAB-0766, Maixin Biotech, Co., Ltd., Fuzhou, China), GSDMD (1:200 dilution, v/v, #DF12275, Affinity Biosciences Ltd., Changzhou, China), Nrf2 (1:200 dilution, v/v, #16396-1-AP, Proteintech Group, Inc.), NF-κB p65 (1:500 dilution, v/v, #10745-1-AP, Proteintech Group, Inc.), p-NF-κB p65 (Ser536) (1:500 dilution, v/v, #AF2006, Affinity Biosciences Ltd.), NLRP3 (1:200 dilution, v/v, #DF7438, Affinity Biosciences Ltd.), ASC (1:200 dilution, v/v, #DF6304, Affinity Biosciences Ltd.), caspase-1 (1:200 dilution, v/v, #3866, Cell Signaling Technology, Inc., Danvers, USA), IL-1β (1:200 dilution, v/v, #12242, Cell Signaling Technology, Inc.) and IL-18 (1:200 dilution, v/v, #DF6252, Affinity Biosciences Ltd.). The sections were subsequently incubated with HRP-conjugated secondary antibodies (#RQ7025, Quanhui Imp & Exp Int’l Co., Ltd., Zhuhai, China) at RT for 30 min and exposed to diaminobenzidine chromogen for 15 sec–3 min. The sections were counterstained with hematoxylin and sealed with neutral balsam. Representative micrographs of the stained tissue were captured with a light microscope. In every stained section, three glomeruli were randomly selected for semiquantitative analysis by using Image-Pro Plus 6.0 software (Media Cybernetics, Rockville, USA). The integrated optical density (IOD) and area of each glomerulus were calculated, and the average IOD/area of all three glomeruli in each section was considered the protein expression level of the sample.

***Oxidative stress analysis and ELISA***

The malondialdehyde (MDA) levels in the renal cortex were assessed based on the thiobarbituric acid method by using an MDA assay kit (#A003-1). Superoxide dismutase (SOD) activity in the renal cortex was assessed based on the water-soluble tetrazolium salt-1 method by using a SOD assay kit (#A001-3). Catalase (CAT) activity in the renal cortex was assessed based on the ammonium molybdate method by using a CAT assay kit (#A007-1-1). γ-Glutamylcysteine synthetase (γ-GCS) activity in the renal cortex was assessed based on the microdetermination of phosphorus method by using a γ-GCS assay kit (#A091-1-1). 8-Isoprostane and 8-hydroxydeoxyguanosine (8-OHdG) levels in the renal cortex were tested by using rat 8-isoprostane and 8-OHdG ELISA kits, respectively (#H100-1-2, #H165-1-2). All kits were purchased from Jiancheng Bioengineering Institute (Nanjing, China). To test serum IL-1β and IL-18 levels, rat IL-1β and IL-18 ELISA kits (#EK0393 and #EK0592, Boster Biological Technology Co. Ltd., Wuhan, China) were used, respectively. Samples for oxidative stress analysis and ELISA were prepared and measured according to the manufacturers’ protocols.

***RT–qPCR***

Fresh glomeruli were isolated from the rat as previously described(15). RNA was extracted from the glomeruli by using an RNAeasy™ Animal RNA Isolation Kit with a Spin Column (#R0026, Beyotime Biotechnology) and reverse transcribed with PrimeScript™ RT Master Mix (#RR036A, Takara Bio, Inc., Shiga, Japan) according to the manufacturers’ protocols. qPCR was conducted by using TB Green® Premix Ex Taq™ II (#RR820A, Takara Bio, Inc.) on a real-time PCR amplification and detection system (LightCycler® 480 II, Roche Diagnostics, Indianapolis, USA). The sequences of the primers were as follows: GSDMD (F: 5’-AGATCGTGGATCATGCCGTC-3’; R: 5’-AGGAGGCAGTAGGGCTTGAA-3’); NLRP3 (F: 5’-CCTTCTGAACCGAGACGTGA-3’; R: 5’-CCAAAGAGGAAGCGTACAACA-3’); ASC (F: 5’-GCTGAGCAGCTGCAAAAGAT-3’; R: 5’-GCAATGAGTGCTTGCCTGTG-3’); caspase-1 (F: 5’-TAGACTACAGATGCCAACCAC-3’; R: 5’-CTTCTTATTGGCATGATTCCC-3’); IL-1β (F: 5’-CAGAAGAATCTAGTTGTCCGT-3’; R: 5’-TGTGCTTCATTCATAAACACT-3’); IL-18 (F: 5’-TATCGACCGAACAGCCAACG-3’; R: 5’-GATAGGGTCACAGCCAGTCC-3’); and GAPDH (F: 5’- GCTCTCTGCTCCTCCCTGTTCT-3’; R: 5’-GGCAACAATGTCCACTTTGTCAC-3’). Relative expression levels of target genes in the different groups were compared and calculated by using the Livak method.

***Western blotting***

Total proteins were isolated from fresh renal cortex tissue by using the Whole Cell Lysis Assay (#KGP250, KeyGEN Biotech, Nanjing, China), and nuclear proteins were isolated from fresh renal cortex tissue by using the Nuclear and Cytoplasmic Protein Extraction Kit (#KGP150, KeyGEN Biotech). Equal amounts of denatured protein mixed with loading buffer were then separated by SDS-PAGE and transferred to PVDF membranes. After being blocked with 5% BSA, the membranes were incubated with the following antibodies at 4 °C overnight: anti-GSDMD (1:1500 dilution, v/v), Nrf2 (1:2000 dilution, v/v), NF-κB p65 (1:1000 dilution, v/v), p-NF-κB p65 (Ser536) (1:1000 dilution, v/v), NLRP3 (1:1000 dilution, v/v), ASC (1:1000 dilution, v/v), caspase-1 (1:1000 dilution, v/v), IL-1β (1:1000 dilution, v/v), IL-18 (1:1000 dilution, v/v), GAPDH (1:3000 dilution, v/v, #CW0100M, Cwbio IT Group, Taizhou, China) and Histone H3 (1:3000 dilution, v/v, #17168-1-AP, Proteintech Group). The membranes were then subsequently incubated with HRP-conjugated secondary antibodies (1:2000 dilution, v/v, #A0216/#A0208, Beyotime Biotechnology) at RT for 2 hr and visualized using an Enhanced Chemiluminescent Kit (#P10200, New Cell & Molecular Biotech Co., Ltd., Suzhou, China). Images were captured by using an automatic chemiluminescence/fluorescence image analysis system (5200 Multi, Tanon Science & Technology Co., Ltd., Shanghai, China). The IOD value of each protein band was calculated by using Image-Pro Plus 6.0 software, and the ratio of the IOD relative to the internal control was considered the protein expression level of the sample.

***Statistical analysis***

At least three biological replicates were used for each experiment, and the statistical analyses were performed using SPSS 20.0 software (IBM Corporation, Armonk, USA). The data are presented as the mean ± standard deviation (SD). ANOVA with LSD-t test (equal variances assumed) or Welch with Dunnett’s T3 test (equal variances not assumed) was used for multiple comparisons among the groups. All tests were two-sided, and *P*<0.05 was considered to indicate statistical significance.

**Figure 1:**
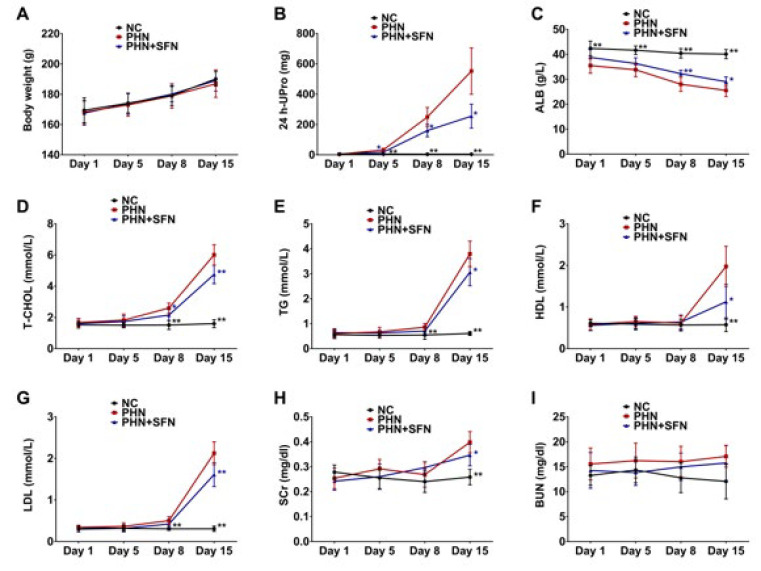
SFN alleviates nephrotic syndrome in PHN rats

Body weight (A), 24 hr-UPro (B), ALB (C), T-CHOL (D), TG (E), HDL (F), LDL (G), SCr (H), and BUN (I) of the rats (n=6) in each group were tested on days 1, 5, 8, and 15 during the observation period. The data are shown as the mean ± SD and were compared to those of the PHN group; *, *P*<0.05; **,* P*<0.01.

SFN: Sulforaphane; PHN: Passive heymann nephritis

**Figure 2:**
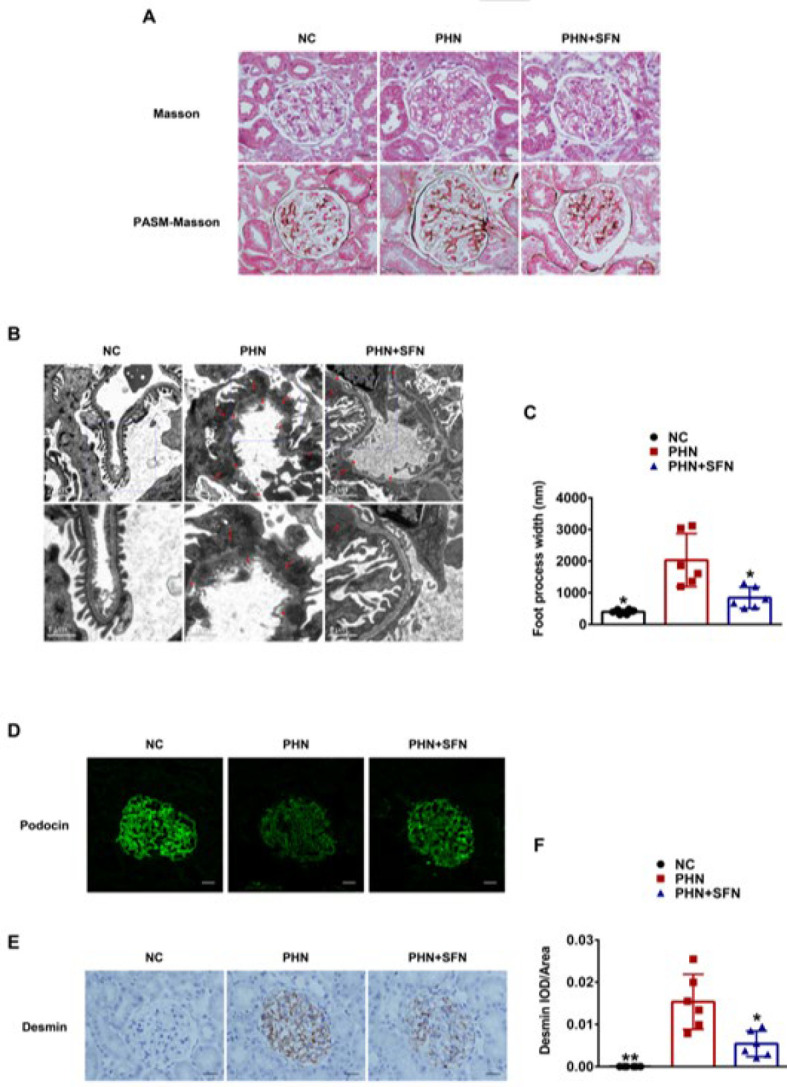
SFN ameliorated podocyte injury in PHN rats

Representative renal Masson and PASM-Masson staining (A) of the rats (n=6) in each group; scale bars=20 μm. (B-C) Representative glomerular TEM images (B) and podocyte foot process width (C) of the rats (n=6) in each group; , subepithelial dense deposit; ,↑ podocyte foot process fusion. Representative renal immunofluorescence staining of podocin (D) of the rats (n=6) in each group; scale bars=20 μm. (E-F) Representative renal immunohistochemical staining of desmin (E) and semiquantification based on the glomerular IOD/area (F) of the rats (n=6) in each group; scale bars=20 μm. The data are shown as the mean ± SD and were compared to those of the PHN group; *, *P*<0.05; **, *P*<0.01.

SFN: Sulforaphane; PHN: Passive heymann nephritis

**Figure 3:**
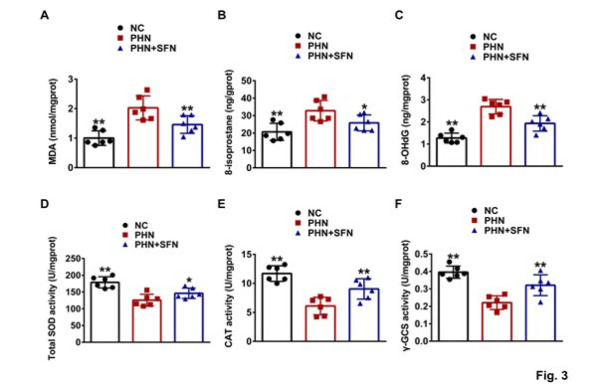
SFN relieved oxidative stress in the kidneys of PHN rats

MDA (A), 8-isoprostane (B), and 8-OHdG (C) in the renal cortex of the rats (n=6) in each group; Activity of total SOD (D), CAT (E), and γ-GCS (F) in the renal cortex of the rats (n=6) in each group. The data are shown as the mean ± SD and were compared to those of the PHN group; *, *P*<0.05; **, *P*<0.01.

SFN: Sulforaphane; PHN: Passive heymann nephritis

**Figure 4:**
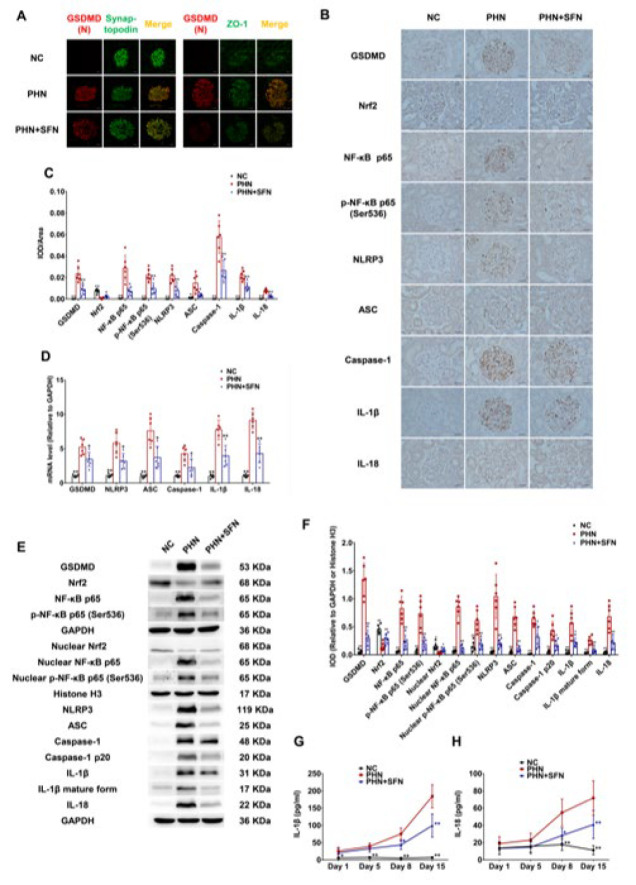
SFN inhibited podocyte pyroptosis in the kidneys of PHN rats

Representative images of renal double immunofluorescence staining for GSDMD(N)/synaptopodin and GSDMD(N)/ZO-1 (A) of the rats (n=6) in each group; scale bars=20 μm. (B-C) Representative renal immunohistochemical staining for GSDMD, Nrf2, NF-κB p65, p-NF-κB p65 (Ser536), NLRP3, ASC, caspase-1, IL-1β, and IL-18 (B) and semiquantification based on the glomerular IOD/area (C) of the rats (n=6) in each group; scale bars=20 μm. Relative mRNA levels of glomerular GSDMD, NLRP3, ASC, caspase-1, IL-1β, and IL-18 (D) of the rats (n=6) in each group. (E-F) Representative renal western blot images of GSDMD, Nrf2, NF-κB p65, p-NF-κB p65 (Ser536), nuclear Nrf2, nuclear NF-κB p65, nuclear p-NF-κB p65 (Ser536), NLRP3, ASC, caspase-1, caspase-1 p20, IL-1β, IL-1β (mature form), IL-18 and the internal control (GAPDH, Histone H3) (E) and semiquantification based on IOD (relative to GAPDH or Histone H3) (F) of the rats (n=6) in each group; Serum IL-1β (G) and IL-18 (H) levels of the rats (n=6) in each group on days 1, 5, 8, and 15 during the observation period. The data are shown as the mean ± SD and were compared to those of the PHN group; *, *P*<0.05; **, *P*<0.01.

SFN: Sulforaphane; PHN: Passive heymann nephritis

## Results

***SFN alleviates nephrotic syndrome in PHN rats***

We first examined the effect of SFN on the clinical manifestation of PHN rats. Similar changes in weight gain were observed in the NC, PHN, and PHN+SFN groups during the 15-day observation period (Figure 1A), suggesting the safety of SFN for the treatment of PHN rats. Typical nephrotic syndrome was observed in PHN rats, including significantly increased levels of 24 hr-UPro, decreased ALB, and hyperlipemia, as indicated by increases in T-CHOL, TG, HDL, and LDL beginning on the 5th day after model establishment compared to those in NC rats (Figure 1B-G). Increases in SCr and uptrend of BUN were also observed on the 15th day in PHN rats (Figure 1H-I), indicating the impairment of renal function. SFN effectively relieved nephrotic syndrome, which was characterized by reduced 24 hr-UPro, T-CHOL, TG, HDL and LDL in the PHN+SFN group and increased ALB levels compared with those in the PHN group after eight days (Figure 1B-G). SFN was also beneficial for maintaining kidney function in PHN rats because of the reductions in SCr levels in the PHN+SFN group on the 15th day (Figure 1H). In brief, these results indicated that SFN could alleviate nephrotic syndrome in PHN rats.

***SFN ameliorated podocyte injury in PHN rats***

Based on the clinical manifestation, we proceeded to explore the protective effect of SFN on podocytes at the histopathological and molecular levels. As shown in Figure 2A, pathological features of MN, such as subepithelial deposits of the fuchsin protein and spike-like changes in the GBM were observed in the glomeruli of PHN rats via Masson and PASM-Masson staining. TEM revealed histopathological characteristics of MN in PHN rats, including glomerular subepithelial dense deposits and widespread foot process fusion of podocytes, as indicated by a substantial increase in foot process width (Figure 2B-C). Podocin, which is the principal member of the integral podocyte slit diaphragm protein family, was significantly decreased and redistributed in the glomeruli of PHN rats compared to those of NC rats, as shown by immunofluorescence staining (Figure 2D). Additionally, desmin, a classic molecular marker of podocyte injury, was increased in the glomeruli of PHN rats (Figure 2E-F). These results showed a clear histopathological and molecular profile of MN and indicated that the podocytes were impaired. SFN significantly ameliorated podocyte injury, as indicated by the decreased foot process width due to the alleviation of foot process fusion (Figure 2B-C), increased expression and normalized distribution of podocin, and decreased expression of desmin in the glomeruli of PHN+SFN rats compared to PHN rats (Figure 2D-F). Collectively, these findings demonstrated that SFN could ameliorate podocyte injury in PHN rats.

***SFN relieved oxidative stress in the kidneys of PHN rats***

We further investigated the mechanisms underlying the renoprotective and podocyte-protective effects of SFN. Oxidative stress, which is closely associated with pyroptosis, was evaluated. As shown in Figure 3A-B, the levels of MDA and 8-isoprostane, which are two reliable markers of lipid peroxidation, were significantly greater in the renal cortex of PHN rats compared to NC rats. The level of 8-OHdG, another marker of oxidative stress that indicates the oxidation of DNA, was also increased (Figure 3C). In contrast, the activities of anti-oxidant enzymes, including total SOD, CAT, and γ-GCS, were lower in the renal cortex of PHN rats than NC rats (Figure 3D-F). SFN exhibited intrarenal anti-oxidant effects, as indicated by decreased MDA, 8-isoprostane, and 8-OHdG levels, and increased total SOD, CAT, and γ-GCS activities in the treatment group (Figure 3A-F). In summary, these results showed that SFN could relieve oxidative stress in the kidneys of PHN rats.

***SFN inhibited podocyte pyroptosis in the kidneys of PHN rats***

We finally examined the effect of SFN on podocyte pyroptosis in the context of intrarenal injury and oxidative stress alleviation. As shown in Figure 4A, significantly greater colocalization of the pyroptosis executor GSDMD(N) with the podocyte marker synaptopodin and the cytomembrane marker ZO-1 was observed in the PHN group than in the NC group, suggesting the activation and membrane translocation of GSDMD and indicating the occurrence of podocyte pyroptosis. Pyroptosis and its potential upstream signaling pathway were activated, as indicated by the marked increase in the expression levels of GSDMD, NF-κB p65, p-NF-κB p65 (Ser536), NLRP3, ASC, caspase-1, IL-1β and IL-18 in the glomeruli and kidney, as shown by immunohistochemical staining, qPCR and western blotting (Figure 4B-F). Activated fragments of caspase-1 and IL-1β, including caspase-1 p20 and the mature form of IL-1β, were also increased in the kidney (Figure 4E-F). Significantly increased levels of IL-1β and IL-18, which are likely derived from pyroptotic podocytes, were also found in the serum of PHN rats beginning eight days after model establishment (Figure 4G-H). Moreover, the potential encounter of the pyroptosis signaling pathway, Nrf2, was down-regulated and insufficient (Figure 4B-C, E-F). These findings validated the occurrence of pyroptosis in the podocytes of PHN rats. SFN effectively inhibited podocyte pyroptosis, as shown by the decreased colocalization of GSDMD(N) with synaptopodin and ZO-1 (Figure 4A); decreased expression levels of glomerular and renal GSDMD, NLRP3, ASC, caspase-1, IL-1β and IL-18 and their activated forms (Figure 4B-F); and decreased release of serum IL-1β and IL-18 in the PHN+SFN group compared to the PHN group (Figure 4G-H). SFN also activated Nrf2 and inhibited NF-κB p65 at overall and activated levels (nuclear translocation) in the glomeruli and kidneys of PHN rats (Figure 4B-C, E-F). Taken together, these results validated the inhibitory effect of SFN on podocyte pyroptosis in PHN rats.

## Discussion

The current study showed that SFN could alleviate MN by inhibiting oxidative stress-associated podocyte pyroptosis. As a gasdermin-dependent and proinflammatory form of programmed cell death, pyroptosis in podocyte-associated kidney diseases is becoming increasingly prominent. In immortalized mouse podocytes, high glucose can induce podocyte pyroptosis by up-regulating NLRP3, ASC, caspase-1, and GSDMD(N), while in the kidneys of diabetic nephropathy mice, markers of pyroptosis are also significantly increased (17). In human conditionally immortalized glomerular podocytes treated with serum from MRL/lpr mice or in the kidneys of MRL/lpr mice, notable increases in and activation of NLRP3-induced pyroptosis signaling were observed, suggesting a role of podocyte pyroptosis in promoting lupus nephritis (18). In podocytes overexpressing the HBx gene, simultaneous up-regulation of pyroptosis-related proteins was observed, suggesting the effect of pyroptosis on podocyte injury in hepatitis B virus-associated glomerulonephritis (19). Our study clearly described the occurrence of podocyte pyroptosis in MN since pyroptosis signaling, ranging from NLRP3 to GSDMD, was increased and concurrently activated in the glomeruli and kidneys of PHN rats. In contrast, the N-terminal fragment of GSDMD, which is the executor of pyroptosis, was increased and translocated to the cytomembrane in podocytes. The increases in serum IL-1β and IL-18 may also be evidence of podocyte pyroptosis in MN. These findings provide important evidence for pyroptosis-targeted treatment of MN in the future.

Oxidative stress is defined as increased production of ROS and a concomitant decrease in the anti-oxidant defense system (20). Dysregulation induced by oxidative stress is crucial in the development of most kidney injury (21-24). In MN, oxidative stress was previously described by Liu *et al.* (25), in which increased levels of MDA and decreased activity of anti-oxidants, including SOD, glutathione, and CAT, were found in the kidneys of PHN rats 37 days after model establishment. Our results revealed similar increases in the levels of MDA and decrease in the activity of total SOD and CAT in the kidneys of PHN rats 15 days after model establishment, suggesting the presence of a persistent milieu of oxidative stress during the development of MN. Additionally, levels of more oxidative stress indicators, including 8-isoprostane, products of arachidonic acid peroxidation by free radicals, and 8-OHdG, products of guanine oxidation from damaged DNA, increased. Moreover, decreased activities of additional anti-oxidases, including γ-GCS, were observed. Our findings further validated the occurrence of intrarenal oxidative stress in MN. Recently, the association between oxidative stress and pyroptosis has gradually become clear. First, the expression, assembly, and activation of the NLRP3 inflammasome can be promoted by ROS. Second, specific Cys residues in GSDMD and possibly other gasdermins can be directly oxidized by ROS (14). Using a complement-induced podocyte damage model, Wang *et al.* (11) found that complement stimulation caused mitochondrial depolarization and ROS production in podocytes while inhibiting ROS reversed complement-induced pyroptosis. The results validate the oxidative stress-mediated podocyte pyroptosis. Our study confirmed and extended Wang *et al.*’s findings since the co-occurrence of oxidative stress and podocyte pyroptosis were also observed in PHN models, suggesting the promoting effect of oxidative stress on podocyte pyroptosis in MN.

SFN is an aliphatic isothiocyanate derived from glucoraphanin that is abundant in the sprouts of cruciferous vegetables. SFN is a key regulator of redox homeostasis that exerts its cytoprotective effects mainly by activating nuclear factor erythroid 2-related factor 2 (Nrf2) and its corresponding anti-oxidant pathway (12). Nrf2 is a master transcription factor that regulates oxidative stress responses. Under unstimulated conditions, Nrf2 is retained in the cytoplasm through an interaction with Kelch-like ECH-associated protein 1 (Keap1), resulting in the proteasomal degradation of Nrf2. Stimuli, including oxidative stress, can disrupt the Nrf2-Keap1 interaction, preventing the degradation of Nrf2 and leading to the stabilization and nuclear translocation of Nrf2, which drives the expression of target genes, including cytoprotective, detoxifying, and anti-oxidant enzymes (26). Activation of Nrf2 can inhibit pyroptosis by suppressing the oxidation of Cys residues of GSDMD(N) or inhibiting NLRP3 inflammasome activity by preventing ROS production (14, 27). Additionally, Nrf2 can repress GSDMD independent of these anti-oxidant effects (28). We observed decreased expression and inactivation of Nrf2 in the glomeruli and kidneys of PHN rats, suggesting a deficiency in renal anti-oxidant capacity in the milieu of MN. SFN effectively stimulates the expression and activation of Nrf2, accompanied by oxidative stress relief and pyroptosis inhibition. In addition, NF-κB, another direct inhibitory target of SFN that is potentially upstream of the pyroptosis signaling pathway and is involved in the pathogenesis of MN (29, 30), was also investigated in our work. As expected, SFN suppressed the up-regulation and activation of NF-κB in the glomeruli and kidneys of PHN rats. Moreover, SFN was also reported to reduce the activation of NLRP3 and inhibit the autoproteolytic activation of caspase-1 and the maturation of IL-1β independent of its Nrf2 activation and anti-oxidant effects, in other words, SFN had the independent anti-pyroptosis effect (31). Therefore, it is reasonable to speculate that the targets associated with oxidative stress and pyroptosis underlying the protective effect of SFN on MN may be multiple. Further investigations on the precise targets and mechanisms of SFN on MN are still needed.

In summary, the current study validated the ameliorative effect of SFN on oxidative stress-associated podocyte pyroptosis through the use of PHN rat models. Nevertheless, this study had certain limitations. First, the causal relationship and the regulatory mechanism between oxidative stress and pyroptosis were insufficiently elaborated by the current data. Second, due to the single observation points, dynamic changes in oxidative stress and pyroptosis in podocytes during the development of MN are still unknown. Despite these limitations, to our knowledge, this is still the first study showing the protective effect of SFN against MN and that the underlying mechanism includes inhibiting oxidative stress-associated podocyte pyroptosis, shedding light on SFN in the treatment of MN in the future.

## Conclusion

SFN could alleviate MN through inhibition of oxidative stress-associated podocyte pyroptosis, making it a promising treatment for MN in the future.

## Data Availability

The datasets used or analyzed during the current study are available from the corresponding author upon reasonable request.

## Declaration

We have not used any AI tools or technologies to prepare this manuscript.
